# Supplementary material for: Causal Relationship between Aging and Anorexia Nervosa: A White-Matter-Microstructure-Mediated Mendelian Randomization Analysis
Source: Biomedicines. 2024 Aug 16;12(8):1874. doi: 10.3390/biomedicines12081874 (PMC11351342; doi:10.3390/biomedicines12081874)
Supplement: Supplementary file 1 [file biomedicines-12-01874-s001.zip › Supplementary Figure/Figure S4/Singe SNP plot of MR analysis for TL to ubm-b-2003.pdf]

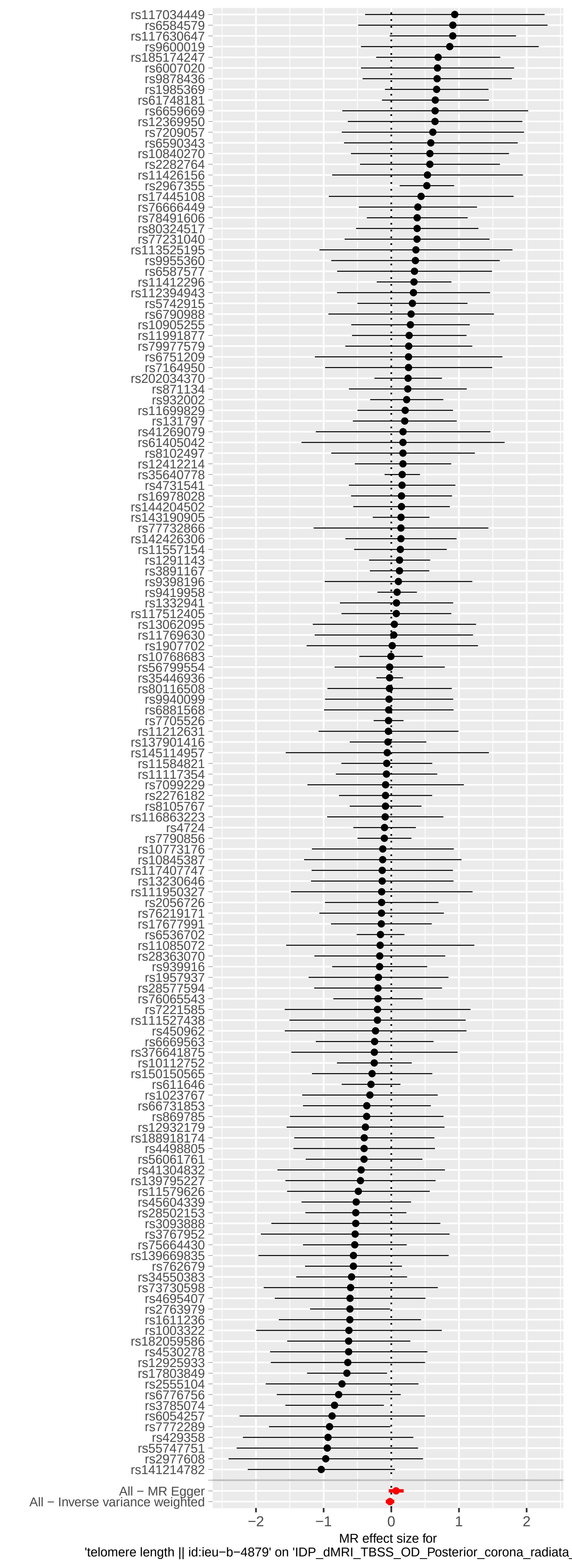
rs117034449
rs6584579
rs117630647
rs9600019
rs185174247
rs6007020
rs9878436
rs1985369
rs61748181
rs6659669
rs12369950
rs7209057
rs6590343
rs10840270
rs2282764
rs11426156
rs2967355
rs17445108
rs76666449
rs78491606
rs80324517
rs77231040
rs113525195
rs9955360
rs6587577
rs11412296
rs112394943
rs5742915
rs6790988
rs10905255
rs11991877
rs79977579
rs6751209
rs7164950
rs202034370
rs871134
rs932002
rs11699829
rs131797
rs41269079
rs61405042
rs8102497
rs12412214
rs35640778
rs4731541
rs16978028
rs144204502
rs143190905
rs77732866
rs142426306
rs11557154
rs1291143
rs3891167
rs9398196
rs9419958
rs1332941
rs117512405
rs13062095
rs11769630
rs1907702
rs10768683
rs56799554
rs35446936
rs80116508
rs9940099
rs6881568
rs7705526
rs11212631
rs137901416
rs145114957
rs11584821
rs11117354
rs7099229
rs2276182
rs8105767
rs116863223
rs4724
rs7790856
rs10773176
rs10845387
rs117407747
rs13230646
rs111950327
rs2056726
rs76219171
rs17677991
rs6536702
rs11085072
rs28363070
rs939916
rs1957937
rs28577594
rs76065543
rs7221585
rs111527438
rs450962
rs6669563
rs376641875
rs10112752
rs150150565
rs611646
rs1023767
rs66731853
rs869785
rs12932179
rs188918174
rs4498805
rs56061761
rs41304832
rs139795227
rs11579626
rs45604339
rs28502153
rs3093888
rs3767952
rs75664430
rs139669835
rs762679
rs34550383
rs73730598
rs4695407
rs2763979
rs1611236
rs1003322
rs182059586
rs4530278
rs12925933
rs17803849
rs2555104
rs6776756
rs3785074
rs6054257
rs7772289
rs429358
rs55747751
rs2977608
rs141214782
All − MR Egger
All − Inverse variance weighted
−2
−1
0
1
2
MR effect size for
'telomere length || id:ieu−b−4879' on 'IDP_dMRI_TBSS_OD_Posterior_corona_radiata_
